# The Role of Emotional and Meta-Emotional Intelligence in Pre-adolescents’ Well-Being and Sociometric Status

**DOI:** 10.3389/fpsyg.2021.749700

**Published:** 2021-09-16

**Authors:** Antonella D’Amico, Alessandro Geraci

**Affiliations:** ^1^Dipartimento di Scienze Psicologiche, Pedagogiche, dell’Esercizio Fisico e della Formazione, Università degli Studi di Palermo, Palermo, Italy; ^2^MetaIntelligenze Onlus, Palermo, Italy

**Keywords:** emotional intelligence, meta-emotional intelligence, well-being, sociometric status, pre-adolescence

## Abstract

The study examined the relationships among emotional and meta-emotional intelligence, well-being, and sociometric status in 105 pre-adolescents. Emotional and meta-emotional intelligence were measured using the Intelligenza Emotiva: Abilità, Credenze e Concetto di Sé Meta-Emotivo (IE-ACCME) test (D’Amico, 2013), allowing to measure ability emotional intelligence (EI), emotional self-concept, meta-emotional knowledge, meta-emotional ability in self-evaluation, and meta-emotional beliefs. Meta-emotional dimensions refer to the awareness of individuals about their emotional abilities and to their beliefs about the functioning of emotions in everyday life. Eudemonic well-being and sociometric status were, respectively, measured using the well-known Psychological Well-Being (PWB) scale by Ryff’s (1989) and registering the levels of acceptance/rejection from peers (Moreno, 1960). Results demonstrated that: pre-adolescents’ meta-emotional beliefs are positively associated to eudemonic well-being: pre-adolescents with higher levels of ability EI, meta-emotional knowledge and meta-emotional self-evaluation are more accepted by others while those that overestimate their emotional abilities are more refused by peers. These results evidence that meta-emotional variables may play a crucial role in well-being and sociometric status, encouraging future studies on this issue.

## Introduction

Emotional intelligence (EI) is an umbrella term that includes different theoretical models and measurement methods (see Zeidner et al., 2008; Brackett et al., 2011) that have been classified in different ways (O’Connor et al., 2019). Under a theoretical point of view, one of the most used classification refers to three main approaches: the ability model (Salovey and Mayer, 1990; Mayer and Salovey, 1997) the trait models (Petrides and Furnham, 2000, 2001) and the mixed models (Goleman, 1995; Bar-On, 1997). Mayer and Salovey (1997) defined ability EI as an interrelated set of cognitive abilities involved in emotional problem solving. Petrides and Furnham (2000, 2001) conceived trait EI as a series of emotion related personality traits. Bar-On (1997) and Goleman (1995; see also Boyatzis et al., 2000; Boyatzis, 2009) conceive EI as a constellation of traits and emotion-related skills or competencies and for this reason it has been defined as mixed EI.

The different theoretical approaches to EI correspond also to different measurement methods: Mayer and Salovey (1997) argued that, since EI is a form of intelligence, it should only be measured by performance test like MSCEIT (Mayer et al., 2002) or STEM and STEU (MacCann and Roberts, 2008) requiring individuals to solve emotional tasks or problems. Petrides and Furnham (2000, 2001), supporting the trait model of EI, adopted self-report methodologies that are widely used in measuring personality traits. Consistent with the mixed models of EI, researchers use mixed methods: Bar-On (1997) made a scale based on self-report, while Sala (2002) created a scale based on both self-report and other-report methodologies. A further group of assessments tools (Schutte et al., 1998; Brackett and Mayer, 2003; Brackett et al., 2006) have been defined as self-reported ability EI (Gutiérrez-Cobo et al., 2016) since they use self-report methodologies but are focused only the Mayer and Salovey ability model of EI and do not include personality traits or competencies related to emotions.

The existence of deep epistemological differences among theoretical frameworks of EI and their relative measurements methods produced a big debate in this area, leading scholars to wonder if EI should be considered as an useful construct or, rather, “more myth than science” (Matthews et al., 2002; Zeidner et al., 2008; Hugh and Evans, 2018).

Nevertheless, scientific literature of the last 20 years demonstrated important associations among EI and other aspects of individuals’ life, although the predictive value of EI in the different dimensions of life also varies according to the type of EI model considered and to the measurement tools used [see reviews on ability EI by Fiori and Vesely-Maillefer (2018); see reviews on trait EI by Pérez-González et al. (2020)].

### Emotional Intelligence, Well-Being, and Sociometric Status

In this paper, our specific interest is about the role of EI in pre-adolescents’ well-being and sociometric status, intended as the level of acceptance/rejection by peers.

Some interesting studies already demonstrated that trait and mixed EI are highly related to well-being (Furnham and Petrides, 2003; Petrides et al., 2007; Di Fabio and Saklofske, 2014; Marjanović and Dimitrijević, 2014; Prado Gascó et al., 2015; Di Fabio and Kenny, 2016; Guerra-Bustamante et al., 2019), both when well-being is conceived in the hedonic perspective, as levels of happiness, pleasure attainment and pain avoidance, or the prevalence of positive affect over negative affect (Diener et al., 1985; Watson et al., 1988), and when it is conceived in the eudemonic perspective, as the level of self-realization and adaptive functioning (Ryff, 1989; Ryff and Keyes, 1995; Ryff and Singer, 2008; Morgan and Farsides, 2009).

On the contrary, results on the relationships among ability EI, hedonic, and eudemonic well-being, are not always consistent. In early adolescents, Zeidner and Olnick-Shemesh (2010) failed to find significant relationships between ability EI and hedonic well-being; moreover, Di Fabio and Saklofske (2014) and Di Fabio and Kenny (2016) evidenced no relationships between ability EI and hedonic nor eudemonic well-being.

In late adolescents or young adults, the relationship between hedonic well-being and ability EI was found only in a study by Extremera et al. (2011) while eudemonic well-being and ability EI were consistently associated in studies by Extremera et al. (2011); Burrus et al. (2012), and Dimitrijević et al. (2018). Rossen and Kranzler (2009) have found also that ability EI was related to eudemonic well-being even after controlling for general cognitive ability and personality factors.

Emotional intelligence is also associated with sociometric status, that has been firstly defined by Moreno (1960) as the level of acceptance and rejection by peers and that is a very important factor for adolescents’ social and psychological development (Rubin et al., 2004). Sociometric status is usually measured by asking people to indicate a few peers in their group that they would choose (level of acceptance) or that they would not choose (level of rejection) for a particular activity or situation. Acceptance and rejection scores, respectively, correspond to the number of people in a group from whom a person is accepted or rejected, while perceived acceptance and rejection scores correspond to the number of people in a group from whom a person thinks he could be accepted or rejected.

Petrides et al. (2006) demonstrated that children with high levels of trait EI that have better sociometric status than others; they are more likely to be seen as leaders, and they are seen as cooperative rather than aggressive and dependent by peers. In a similar study, also Mavroveli et al. (2007) showed that trait EI was positively related to peer-rated social competence.

Zavala et al. (2008) assessed mixed-EI (Bar-On and Parker, 2000) in a group of adolescents selected for their high sociometric status and in a control “natural” group. Results showed that adolescents in the higher sociometric status group obtained higher scores than control group in mixed EI. Similarly, Fotopoulou et al. (2019) found that trait EI was related to better social cohesion, assessed by the peer nomination technique.

Other studies confirmed the relationship between EI and sociometric status, evidencing also that the strength of the association is highly influenced by sex of participants. Mabekoje and Ogunyemi (2003) measured the sociometric status within the classroom and the self-reported ability EI (Schutte et al., 1998) in a group of 17 years old adolescents, demonstrating that female students with high sociometric status had higher EI scores than female students with low sociometric status. On the contrary, male students with low sociometric status had higher EI scores than male students with high sociometric status. Also, Andrei et al. (2015), in a sample of Italian children and early adolescents, found that trait EI is positively related to the level of acceptance and negatively related to self-perceived rejection, but this relationship is mediated by sex. Indeed, only females that show high levels of trait EI obtain higher scores in social acceptance; similarly, only girls that show low levels of trait EI obtain higher scores in self-perceived rejection.

The relationship between ability EI and sociometric status, have not been investigated yet, at least not under the traditional ability EI theoretical framework and nor in adolescents or even early adults. However, two studies measuring emotional performances and sociometric status in children reported interesting results: Edwards et al. (1984) found that children with higher sociometric status scores obtained significantly higher scores in a task of recognition of emotion in child photographs compared to children with lower sociometric scores; Sastre et al. (2019) demonstrated that a group of children defined as “sensitive” for their abilities in perceiving and understanding emotions, in a test based on the interpretation of cinema scenes, were considered the best friends by their classmates more often than others.

In conclusion, scientific literature about EI and well-being demonstrated that there is an important association between the two constructs, even if its size may vary depending on the specific theoretical models of EI and well-being considered, and also on the age of participants. In particular, only trait EI but not ability EI seems to be associated with well-being in early adolescents.

Similarly, studies focusing on sociometric status demonstrated that trait EI, mixed EI, and self-reported ability EI, with some sex-related differences, are associated to social acceptance. As for ability EI, further studies conducted with adolescents should be done in order to shed light on its relationship with sociometric status.

Finally, we examined the scientific literature about the association between well-being and sociometric status. Considering the importance of peers during adolescence, we expected indeed that sociometric status influences well-being and vice-versa. However, to the best of our knowledge, only Anderson et al. (2012) addressed this issue in a study involving young adults, demonstrating that sociometric status predicts hedonic well-being, but only marginally eudemonic well-being. Thus, a new study investigating such variables in pre-adolescents is useful in order to give new empirical evidence to this unexplored research area.

### Adding Meta-Emotional Dimensions to EI

The interesting results described so far in scientific literature encouraged us to conduct a new study exploring the relationships among EI, well-being, and sociometric status. However, we were also interested at exploring how well-being and sociometric status are related to a series of meta-emotional dimensions of EI that are measured using the IE-ACCME test Intelligenza Emotiva: Abilità, Credenze e Concetto di Sé Meta-Emotivo (D’Amico, 2013, 2018; D’Amico and Guastaferro, 2017).

The rationale for developing the IE-ACCME test need to be outlined, since it results from a critical analysis of scientific literature about individual differences in all forms of EI studied so far (ability, trait, mixed, and self-perceived EI).

We already described the deep differences among measurement tools used for measuring ability, trait, mixed, and self-reported ability EI, and we know that different tools of EI have different predictive validity toward other aspects of individual life. It is fair to note that ability and trait measures of EI show also problems of convergent validity. Brackett and Mayer (2003) demonstrated that MSCEIT scores are weakly correlated with both Bar-On (1997) scale and Schutte et al. (1998) scale scores, even if the latter is based on the Mayer and Salovey model. A similar result was found by Brackett et al. (2006) in a study were MSCEIT scores were compared to a self-report scale developed by the authors for the scope of the study and based on Mayer and Salovey model.

Brackett et al. (2006) discussed the low concordance between performance-based and self-reports individuals’ scores claiming that perceived measures of EI might be inaccurate for several reasons: social desirability response may influence answers (Paulhus, 1991); individuals with low ability EI may lack of the meta-cognitive skills to report on their EI, and thus report level of EI that are quite different from their actual abilities. For these reasons Brackett et al. (2006), affirmed that performance-based and self-reports “are most likely tapping into different mental processes” (p. 784) and that only performance-based measures should be considered as measures of EI.

D’Amico (2013, 2018), however, stated that both self-concept about one’s own abilities and actual abilities in the emotional field are important for individuals’ emotional life. Indeed, ability EI may drive people to perceive, use, understand, and manage emotions and action, but self-concept about one’s own emotional abilities, even when not corresponding to actual abilities, may drive individuals’ behavior and choices. For these reasons, D’Amico (2013) developed the IE-ACCME test including among tools both an emotional self-concept scale requiring to self-report one’s own emotional abilities in everyday life, and an emotional ability scale requiring to solve emotional performance tasks. Not surprisingly, even if the two scales share the same theoretical model and the same factorial structure, in standardizations study of IE-ACCME total scores of emotional self-concept scale and emotional ability scale showed no correlation each-others (*r* = 0.04).

In order to explain the discordances among self-reported and emotional abilities, D’Amico (2013, 2018) referred to one of the most important aspects of metacognition: the knowledge and self-awareness of one’s own abilities (Flavell, 1979). D’Amico (2018) thus defined the concordance between self-reported and emotional abilities as meta-emotional knowledge: poor meta-emotional knowledge may be due to overestimation errors (when emotional self-concept scores are higher than emotional ability scores) or underestimating errors (when emotional self-concept scores are lower than emotional ability scores). In author perspective, poor meta-emotional knowledge may be dangerous for emotional life both when it leads to overestimation and underestimation (D’Amico, 2018). Indeed, the overestimation of one’s emotional abilities might lead adolescents to copy with situation they are not able to manage; underestimation of their emotional abilities might lead them to avoid those situations that they could be able to front, reducing the experiences of success. For all these reasons, meta-emotional knowledge is measured both considering its size but also the direction of estimation errors.

Another important aspect of metacognition refers to the ability in self-evaluating one’s own performance. Very often, children and adolescent base their self-evaluations on results they achieve at school or in other contexts. A student generally says that he/her is not good in math since he/she obtained a low grade. External feedbacks on specific tasks are also very important since they help people to develop focused strategies for improving their performances. In the field of emotions, however, it is not so simple to self-evaluate one’s own performance because, as also claimed by Brackett et al. (2006), in everyday life, neither academic nor professional settings, give people clear feedback about the efficacy of their behaviors and choices in the emotional field. For this reason, D’Amico (2013) included a self-evaluation question after each task of the emotional ability scale of the IE-ACCME test. Size and direction of discrepancies between adolescents’ self-evaluation of performance in each the emotional ability scale and their actual results in the same scale allow to measure meta-emotional ability in self-evaluation. This meta-emotional dimension is not overlapping with meta-emotional knowledge. In fact, for people and presumably even more for adolescents, self-evaluating one’s own perceived performance in a specific emotional task could be a simpler and more circumscribed task than self-evaluating one’s own emotional abilities in daily or typical situations. Self-evaluation, indeed, resulted not associated to emotional ability (*r* = 0.09), demonstrating that also low meta-emotional ability in self-evaluation is very common among adolescents (D’Amico, 2013).

Finally, a further important aspect of metacognition refers to belief’s system of individuals: thinking and behaviors of people are guided by their beliefs and convictions about particular phenomena (i.e., Dweck, 1999). In recent years, there has been a growing interest also toward the beliefs about emotions, since they are considered important for emotion regulation: for example, Tamir et al. (2007) evidenced that people believing that emotions are uncontrollable show low emotion regulation self-efficacy, less usage of adaptive regulation strategies, poorer social adjustment, and more severe mental health symptoms. A recent review by Ford and Gross (2019) provides a detailed insight into this interesting area of research, and Capobianco et al. (2020) included metacognitive beliefs among the best predictors of anxiety and depression.

D’Amico (2013, 2018) found it useful to include beliefs about emotions among the meta-emotional dimensions, since people who own an implicit belief system consistent with current scientific knowledge on EI are more likely to show also higher levels of ability EI. On the other hand, all psychologist and educators know that the first step of every program for the development of EI is to explain to people that, for instance, also unpleasant emotions may be useful or that emotions are closely associated to physical sensations, and so on. However, beliefs about the different aspects of EI have never been investigated before. For this reason, D’Amico (2013) included in the IE-ACCME test a meta-emotional belief’s questionnaire investigating the adolescents’ beliefs about the perception, use, understanding, and management of emotions. As expected, in the IE-ACCME standardizations study, the total meta-emotional belief score was positively related to the total emotional ability scores (*r* = 0.31), demonstrating that adolescents who owned an adequate belief system about the role of emotions in daily life had higher levels of EI (D’Amico, 2013) and *vice versa*.

In conclusion, emotional and meta-emotional intelligence have not to be considered as opposite but rather complementary constructs. In this new framework, the Mayer and Salovey concept of EI as an ability belonging to the domain of cognitive abilities (MacCann et al., 2014) is preserved; at the same time, the measurement of ability EI is enriched by a series of measures allowing to understand, also, adolescents’ self-concept toward their EI, to what extent they are aware of their ability EI and, in general, which beliefs about EI drive their thinking and their behaviors.

### The Present Study

The specific aim of this study is to investigate if pre-adolescents with high levels of emotional and meta-emotional intelligence experience also high level of well-being, and if they are more accepted and less rejected by peers.

For this purpose, we examined to what extent ability EI, emotional self-concept, meta-emotional knowledge, meta-emotional self-evaluation, and meta-emotional beliefs are associated to eudemonic well-being and sociometric status in a population of students of lower secondary-school.

Considering the results of previous scientific literature that have been described before, we expected to find positive association among the investigated variables.

Regarding well-being, we decided to focus on eudemonic well-being since results from previous literature demonstrated more association between this specific aspect of well-being and EI, rather than between hedonic well-being and EI. These studies demonstrated also that adolescents show stronger relationships between eudemonic well-being and trait or mixed EI, rather than with ability EI. Thus, even if these results have been found using different measurement tools or also different theoretical frameworks, we expected to find more association between emotional self-concept and eudemonic well-being, rather than between ability EI and eudemonic well-being.

Even in the case of sociometric status, our expectation about its positive association with ability EI and emotional self-concept, was based on studies using different measurement tools. Moreover, the association of ability EI and sociometric status has never been explored in pre-adolescents.

As for the relationships among meta-emotional dimensions, well-being and sociometric status, there are no previous studies about this issue. However, we hypothesized that those pre-adolescents with higher levels of meta-emotional knowledge and/or meta-emotional self-evaluation, being more aware of their emotional abilities, could experience higher well-being and own higher sociometric status than others. For pre-adolescents with poor meta-emotional knowledge and/or poor ability in meta-emotional self-evaluation, we were also very curious to know which type of esteem error (overestimation vs. underestimation) could negatively influence eudemonic well-being and sociometric status.

Finally, we hypothesized that owning meta-emotional beliefs that are consistent with what current theories and empirical evidences about EI demonstrated, could be positively associated to emotional ability, eudemonic well-being, and sociometric status.

## Materials and Methods

We used descriptive, group comparison, and correlational methods to achieve the research goals. More details are reported in section “Data Analyses.”.

### Participants and Procedures

This research involved a not random sample composed of 105 students (55 females and 50 males), between 10 and 16 years (*M* = 12 years and 6 months; SD = 15.27 months). They attended six classes (two for each grade) of an Italian secondary lower school in metropolitan area. The classes that attended to the research were selected by the school’s principal, class-size ranged from 12 to 20 students and all children in the six classes were included in the study.

Usually, Italian secondary lower school is attended by students between the age of 11 and 14, but some of the students were of 15 and 16 years of age since they failed one or two school years. Mean age of the female group is 12 years and 6 months (SD = 15.08 months) whereas mean age the male group is 12 years and 7 months (SD = 15.59 months).

All students completed the paper–pencil tests and scales described below at school and in two separate collective sessions of about 1 h each. During the first session they completed the IE-ACCME test, and in the second session they completed both the well-being scale and the sociometric task.

Parents of all adolescents involved in the study signed an informed consent. In order to ensured anonymity and confidentiality of gathered data, each student was asked to write in the front page of each test only his/her sex and age and an assigned numerical code that was used to match the scores in the different tasks. No information about individual scores of each student was shared with teachers or parents, nor among students, and all data were only used in aggregate form.

### Measures

#### Emotional and Meta-Emotional Intelligence

Emotional and meta-emotional intelligence of participants was measured using the multi-trait and multi-method tool IE-ACCME (D’Amico, 2013). As already described, the IE-ACCME is an Italian original test based on the Mayer and Salovey’s (1997) four-branch theoretical model that is not designed only to measure ability EI. Rather, it uses four different tools for calculating scores in: (1) ability EI; (2) emotional self-concept; (3) meta-emotional knowledge; (4) meta-emotional ability in self-evaluation; (5) meta-emotional beliefs.

The first tool is a questionnaire exploring the meta-emotional beliefs of adolescents (CE scale). It includes 16 items with 5-point Likert scale ranging from 0 “not true” to 4 “definitely true” that explore individuals’ beliefs about perception, facilitation, understanding, and management of emotions. After validation, however, only eight items, that explained the 60.2% of variance and focus on the four branches and eight tasks of EI, were selected for computing the CE score. The CE score, thus, represents the degree to which people believes that each aspect of emotion included in the EI ability-based model is important and influences everyday life (i.e., if they believe that sensations produce emotions, that emotions can facilitate thinking, that emotions may be blended each-others, or that emotions can be regulated). One of the items is, for instance, “Complex feelings like love or friendship arise from a mixture of many emotions.”

The second tool is a self-report questionnaire exploring the emotional self-concept (CME scale), i.e., the self-perceived ability in perception, facilitation, understanding, and management of emotions. CME scale includes 20 items with 5-point Likert scale ranging from 0 “not true” to 4 “definitely true.” Even in this case, validation procedure revealed that a solution with eight items, focusing on the four branches and eight tasks of EI, explained the 60.54% of variance and were then selected for computing the CME score. Items ask people to evaluate their emotional abilities in everyday situation (e.g., “I am able to identify the emotions that derive from particular physical sensations”). The CME score represents the degree to which people consider themselves to be able in perceiving, using, understanding, or managing emotions in everyday life.

The third tool is a maximum performance test (AE scale) used for assessing ability EI. The AE scale is similar but not overlapping to MSCEIT (Mayer et al., 2002) and includes eight tasks grouped in four branches: (1) perception of emotions (faces and pictures); (2) facilitation of emotions in cognitive processes (use and sensations); (3) understanding of emotions (blends and transformations); (4) management of emotions (personal and interpersonal management). All AE scale uses the consensus scoring method (Mayer et al., 2002).

The fourth tool is a self-rating about performance scale (AP). After each one of the eight ability EI task, adolescents are requested to self-rate their performance in the task with a 6-point Likert scale ranging from 0 “not able” to 5 “very able.”

Standardization and validation of the IE-ACCME test was performed on 1.084 Italian adolescents: 526 males and 558 females, between 10 and 19 years of age. Structural validation confirmed trough explorative and confirmatory factorial analyses that all IE-ACCME scale reflect Mayer and Salovey’s (1997) four-branch and eight tasks structural model. However, as discussed before, scores of CE, CME, AE, and AP are not or very slightly correlated each-others, indicating that they measure different processes of emotional sphere. All scales present acceptable test-retest reliabilities: (test-retest: CE, *r* = 0.43, *p* < 0.001; CME, *r* = 0.76, *p* < 0.001; AE, *r* = 0.44, *p* < 001; AP, *r* = 0.55, *p* < 001). Total AE score, presents also a good split-half reliability (=0.86). Cronbach alpha was not computed for the IE-ACCME total scores, due to the small number of items in the CE, CME, and AP scales (8), and because the items in the total AE scale are rather heterogeneous (D’Amico, 2013).

All scores of CE, CME, AE, and AP are expressed as standardized scores with mean = 100 and standard deviation = 15. This allows also to compare them in order to obtain a complete individuals’ profile and to compute scores in meta-emotional knowledge and meta-emotional self-evaluation.

The meta-emotional knowledge score corresponds to the discrepancy between CME and AE and it indicates to which degree the performance of participants in the ability test corresponds to their emotional self-concept in everyday life. Importantly, in order to take into account individual differences in ability EI, the difference among CME and AE is weighed on the AE score; thus, the meta-emotional knowledge score corresponds to (CME-AE)/AE. Two types of meta-emotional knowledge scores have been computed: the first (CMetaAbs) corresponds to the absolute discrepancy value: the higher is the score, the lower is the meta-emotional knowledge. The second (CMetaRel) is computed in relative values and allows understanding if respondents tend to overestimate (positive score) or underestimate (negative score) their emotional abilities in everyday life.

The meta-emotional self-evaluation ability corresponds to the discrepancy between AP and AE score, and also in this case it is weighed on the AE score. Meta-emotional self-evaluation ability indicates to which degree the performance of participants in the ability test corresponds to their self-evaluation of performance after each task. Again, the meta-emotional self-evaluation ability score (AVMetaAbs) corresponds to the absolute discrepancy value: the higher is the score the lower is the meta-emotional self-evaluation ability. The meta-emotional self-evaluation ability score in relative values (AVMetaRel) allows understanding if respondents tend to overestimate (positive score) or underestimate (negative score) their performance in testing situation.

In conclusion, we used seven IE-ACCME total scores for each participant, such as: (1) standardized score of meta-emotional beliefs (CE); (2) standardized score of emotional ability (AE); (3) standardized score of emotional self-concept (CME); (4–5) weighted scores of meta-emotional knowledge in absolute and relative values (CMetaAbs total and CMetaRel); (6–7) weighted scores of meta-emotional self-evaluation in absolute and relative values (AVMetaAbs and AVMetaRel).

#### Well-Being

The Italian version by Ruini et al. (2003) of the Psychological Well-Being scale (PWB; Ryff, 1989; Ryff and Keyes, 1995) was used for assessing eudemonic well-being of participants. PWB consists of six sub-scales measuring six dimensions of well-being: self-acceptance, positive relations with others, autonomy, environmental mastery, purpose in life, and personal growth.

The Self-Acceptance sub-scale assesses one’s attitude and perception toward oneself (e.g., “I like most part of my personality”); the Positive Relations with Others sub-scale assesses if one is having close and warm relationships with others (e.g., “Maintaining close relationships has been difficult and frustrating for me”); the Autonomy sub-scale assesses one’s ability to make personal and independent decisions (e.g., “I tend to be influenced by people with strong opinions”); the Environmental Mastery sub-scale assesses one’s ability to manage and change the environment (e.g., “In general, I feel I am in charge of the situations in which I live”); the Purpose in Life sub-scale assesses the sense of having goals and meaningfulness in life (e.g., “I enjoy making plan for the future and working to make them a reality”); the Personal Growth scale assesses one’s feeling of incessant changing and development (e.g., “For me, life has been a continuous process of learning, changing, and growth”). We used the Italian short version of the scale (18 items), translated, and validated by Ruini et al. (2003). In the 18-items version, each of the six dimensions is explored by 3 items. Participants were presented with all the items and were asked to answer to each item using a 5-point Likert scale (1 = completely disagree; 5 = completely agree). Some items are reversed before to compute the final raw score used in the following analyses. Thus, the higher the score, the higher the level of well-being.

#### Sociometric Status

To investigate social relationships among adolescents and pre-adolescents in the same school class, we used a method based on Moreno’s (1960) sociometric theory. We presented to participants four-coupled questions. The first couple explored the affective dimensions: participants were asked to choose, among peers in the classroom, those that they would like to spend free time with (affective acceptance), or those that they would not like to spend free time with (affective rejection). The second couple explored the schoolwork dimensions: participants were asked to choose, in the classroom, those schoolmates that they would like to make schoolwork with (schoolwork acceptance), or those that they would not like to make schoolwork with (schoolwork rejection). For each of the four questions, participants had to indicate the names of three classmates giving them an order of priority (i.e., classmate 1 = first choice; classmate 2 = second choice; classmate 3 = third choice). Thus, when a classmate was chosen/rejected as first one, he/her received a score of 3, when he/her was chosen/rejected as the second, the assigned score was 2, when he/her was chosen as the third, the assigned score was 1. If a classmate was not chosen/rejected at all, the assigned score was equal to 0.

Each participant obtained four scores (affective acceptance; affective rejection; schoolwork acceptance; and schoolwork rejection), corresponding to the sum of values for each acceptance and rejection nomination in the affective and schoolwork situations. Since the score of each participant could also vary according to the size of the class to which participants belong to, the total score of each participant was weighted dividing it by the number of their classmates. The four score where then averaged in order to compute two final raw scores used in the following analyses, and corresponding to the total score of Acceptance and the total score of Rejection. The higher the score the higher the level of Acceptance or Rejection by peers.

#### Data Analyses

We computed descriptive statistics with the aim to overview the distribution of variables in the sample. A series of *t*-test have also been performed to check if there were significant sex differences.

To examine the correlations and the predictive relationships among the study variables, a series of correlational and regression analyses were carried out. Data analyses were performed using the SPSS software package.

## Results

### Descriptive Statistics and Sex Differences

Table 1 presents means, standard deviations, skewness, and kurtosis values and *t*-test results for all the study variables. Skewness and kurtosis values for all scales are acceptable, except than for PWB. Analysis of descriptive data revealed also that there is an important difference among the standardized scores of CME, AE, and AP. In particular, score on AE scale is significantly lower than score in CME (*t* = 8.09, *p* < 0.001) as well as score in AP (*t* = 8.95, *p* < 0.001) demonstrating that, in general, the self-rating of abilities in everyday life or in testing situation is higher than the score obtained in the ability test.

**TABLE 1:** Descriptive statistics and mean differences between males and females for IE-ACCME test, Psychological Well-Being, and Sociogram scores.

							**Males**		**Females**		
**Scale**	**Min**	**Max**	***M***	**SD**	**Skewness**	**Kurtosis**	***M***	**SD**	***M***	**SD**	***t*-Test^*a*^**
CE	53.29	133.43	94.07	18.30	0.03	−0.46	94.59	19.21	93.61	17.59	0.27
CME	60.95	133.03	100.03	14.97	−0.01	−0.49	102.21	15.17	98.06	14.64	1.43
AE	53.29	124.84	82.83	15.92	0.47	0.06	81.58	13.23	83.97	18.06	−0.77
AP	65.19	137.29	102.29	17.18	0.24	−0.68	101.93	17.16	102.23	17.36	0.09
CMetaAbs	0.01	1.25	0.31	0.24	1.41	2.53	0.29	0.25	0.32	0.24	−0.52
CMetaRel	−0.36	1.25	0.25	0.30	0.63	0.82	0.28	0.26	0.22	0.33	0.96
AVMetaAbs	0.00	1.09	0.32	0.25	0.81	0.07	0.32	0.23	0.31	0.27	0.10
AVMetaRel	−0.27	1.09	0.27	0.30	0.37	−0.38	0.28	0.28	0.27	0.32	0.24
PWB	31	74	57.90	6.39	−0.75	2.46	58.71	5.50	57.18	7.06	1.22
Acceptance	0	2.25	0.68	0.45	0.69	0.44	0.63	0.39	0.72	0.50	−1.06
Rejection	0	2.81	0.60	0.60	1.70	2.98	0.72	0.66	0.49	0.53	1.93

*CE, beliefs about emotions; CME, self-concept about emotional abilities; AE, ability-based emotional intelligence; AP, self-rating about performance; CMetaAbs, meta-emotional knowledge absolute value; CMetaRel, meta-emotional knowledge relative value; AVMetaAbs, meta-emotional self-evaluation absolute value; AVMetaRel, meta-emotional self-evaluation relative value; PWB, Psychological Well-Being. ^a^df = 103.*

Table 1 presents also means divided by sex and *t*-test results. Boys report slightly higher scores in emotional self-concept (boys = 102.21; girls = 98.06) and obtain also a higher mean score of rejection than girls (boys = 0.72; girls = 0.49). However, *t*-tests revealed that such differences have not statistical significance. Thus, sex differences have not been further considered in the following analyses.

### Relationships Among Emotional and Meta-Emotional Intelligence, Psychological Well-Being, and Sociometric Status

Table 2 presents correlation analyses among all considered IE-ACCME scores, PWB scale, and sociogram scores.

**TABLE 2:** Correlations among scores of IE-ACCME test, Psychological Well-Being, and Sociogram.

	**1**	**2**	**3**	**4**	**5**	**6**	**7**	**8**	**9**	**10**	**11**
1. CE	–										
2. CME	0.10	–									
3. AE	0.23*	0.01	–								
3. AP	0.08	0.31**	0.12	–							
5. CMetaAbs	–0.15	0.53**	–0.58**	0.07	–						
6. CMetaRel	–0.13	0.61**	–0.75**	0.08	0.90**	–					
7. AVMetaAbs	–0.18	0.17	–0.60**	0.53**	0.56**	0.60**	–				
8. AVMetaRel	–0.16	0.18	–0.70**	0.60**	0.56**	0.66**	0.92**	–			
9. PWB	0.25*	0.09	0.16	0.09	–0.08	–0.09	–0.06	–0.07	–		
10. Acceptance	0.12	–0.01	0.41**	–0.01	–0.20*	–0.32**	–0.22*	–0.32**	0.21*	–	
11. Rejection	–0.09	–0.03	–0.20*	0.17	0.10	0.14	0.29**	0.29**	–0.20*	–0.34**	–

*p < 0.05, **p < 0.01.

Regarding the intercorrelations among the IE-ACCME scores, results demonstrated that, similarly to what found in the IE-ACCME standardization sample: meta-emotional belief is associated with ability EI (CE vs. AE: *r* = 0.23, *p* < 0.05) whereas emotional self-concept is not related to meta-emotional beliefs nor to ability EI. The significant correlations among IE-ACCME meta-emotional variables with AE or CME scores are not relevant since they are computed starting from the same scale scores and are partially overlapping.

Concerning the relationship among EI variables and eudemonic well-being, only meta-emotional belief’s score results positively associated to PWB score (CE and PWB score: *r* = 0.25, *p* < 0.05). This result demonstrates that adolescents owning a beliefs system toward emotions that is consistent with ability model of EI, experience higher level of eudemonic well-being.

Other interesting results refers to sociometric status. In this case, results showed significant and positive association between acceptance and ability EI (*r* = 0.41, *p* < 0.01), and a negative association between rejection and ability EI (*r* = −0.20, *p* < 0.05). These results demonstrate that adolescents with higher levels of EI are more accepted and less refused by peers. Moreover, acceptance is associated with all scores of meta-emotional knowledge and meta-emotional self-evaluation demonstrating that, independently from adolescents’ level of ability EI, those that are aware of their abilities in everyday life and in testing situation are more accepted by others. Notably, the negative correlations are higher for both meta-emotional knowledge and meta-emotional self-evaluation when they are expressed in relative values. This indicate that, in general, underestimating own emotional abilities produce more acceptation by peers.

In the case of rejection, only meta-emotional self-evaluation is associated with rejection, both when it is computed in absolute (*r* = 0.29, *p* < 0.01) and in relative value (*r* = 0.29, *p* < 0.01). These results demonstrate that, independently from adolescents’ level of ability EI, those that are not able to evaluate their performance in the ability test, in particular when they overestimate their performances, are more refused by others.

Three stepwise regression analyses were then performed with the aim to investigate which one of the IE-ACCME test scores is the best predictor of eudemonic well-being and sociometric status. In each stepwise regression, the scores of eudemonic well-being, acceptance, and rejection were the criterion variables, while all the seven IE-ACCME scores considered in this study entered as predictor variables.

Results (see Table 3) showed that eudemonic well-being was best predicted by meta-emotional beliefs (β = 0.25, *p* < 0.05; *R*^2^ = 0.06), whereas acceptation was predicted by ability EI (β = 0.41, *p* < 0.001; *R*^2^ = 0.17), and rejection by meta-emotional self-evaluation expressed in absolute values (β = 0.29, *p* < 0.01; *R*^2^ = 0.08).

**TABLE 3:** Stepwise regression analyses results.

**Predictors**	**Psychological Well-Being**					
	***B***	**SE**	**β**	***t***	***F***	***R* ^2^**
Model 1 (step 1)					6.71^*^	0.06
CE	0.09	0.03	0.25	2.59^*^		

**Predictors**	**Acceptance**					
	***B***	**SE**	**β**	***t***	***F***	***R* ^2^**

Model 1 (step 1)					20.82^***^	0.17
AE	0.01	0.00	0.41	4.56^***^		

**Predictors**	**Rejection**					
	***B***	**SE**	**β**	***t***	***F***	***R* ^2^**

Model 1 (step 1)					9.38^**^	0.08
AVMetaAbs	0.69	0.22	0.29	3.06^**^		

*For each analysis, all IE-ACCME score considered were entered as predictors.*

^*^p < 0.05, ^**^p < 0.01, ^***^p < 0.001.

*B, unstandardized estimate; SE, standard error; β, standardized value.*

A further interesting result refers to the association among eudemonic well-being and sociometric status. As expected, analysis of correlations revealed that eudemonic well-being is positively related to acceptation (*r* = 0.21, *p* < 0.05) and negatively related to rejection (*r* = −0.20, *p* < 0.05). Thus, in our sample, pre-adolescents that are more accepted and less rejected by peers report higher levels of well-being.

## Discussion

In conclusion, results of the present study allowed to obtain different interesting and innovative results.

The first group of results pertains the emotional and meta-emotional profiles of pre-adolescents involved in the study. A simple check of scores in the various IE-ACCME scale demonstrated very clearly that the self-concept of adolescents about their emotional abilities and the self-evaluation of their performance not always corresponds to their abilities as measured in the ability test. Rather, in our group of participants there is a general tendency to overestimate their own emotional abilities, as demonstrated by the general higher score in emotional self-concept and in self-evaluation of performance than in the ability test, as well as by the positive mean scores in meta-emotional knowledge and meta-emotional self-evaluation. These results demonstrates that pre-adolescents in our group have a general poor meta-cognitive awareness of their emotional abilities and that their meta-emotional knowledge and meta-emotional self-evaluation are biased by overestimation.

This result is probably also due to the young age of participants. During standardization of IE-ACCME, indeed, it was observed that the scores in AE increased between 11 and 19 years of age (D’Amico, 2013), while the scores in CME and AP where not, and some of the CME and AP subdimensions were also inversely correlated to the age of participants, decreasing in older adolescents. On the other hand, it is well known that the developmental trend of all meta-cognitive abilities is not complete before adolescence and over (Weil et al., 2013), and many authors state that, in general, the use of self-assessment in young adolescents may be unreliable (Fan et al., 2006).

Relationships among meta-emotional variables, however, demonstrate that those pre adolescents with high level of meta-emotional self-evaluation abilities (i.e., that are reliable in their self-evaluations) show also high scores in meta-emotional beliefs and, thus, are more attentive, sensitive, and aware of the importance of emotions in everyday life.

The second group of results pertains the relationships between EI and well-being: we failed to find strong evidence for an association both when we investigated the relationship between self-concept of EI and well-being, and when we investigated the relationship between ability EI and well-being.

These two results could depend on two different reasons: the first is that the previous study on this issue involving adolescents evidenced an association between well-being and trait or mixed EI (Furnham and Petrides, 2003; Petrides et al., 2007; Di Fabio and Saklofske, 2014; Marjanović and Dimitrijević, 2014; Prado Gascó et al., 2015; Di Fabio and Kenny, 2016; Guerra-Bustamante et al., 2019), while the emotional self-concept scale used in the present study focuses only on the self-report of emotional abilities and not of other emotion-related personality traits. The second reason could be the age of participants: indeed, an association between well-being and ability EI, was not found in adolescents (Zeidner and Olnick-Shemesh, 2010; Di Fabio and Kenny, 2016) but only in young adults (Rossen and Kranzler, 2009; Extremera et al., 2011; Burrus et al., 2012; Dimitrijević et al., 2018).

Thus, we could guess that pre-adolescents and adolescents are too young to use their emotional abilities for improving their well-being, and that these domains are still independents at this stage of life.

It is interesting, however, that we found an association between well-being and meta-emotional beliefs, since it confirms our hypothesis that owning a beliefs’ system that consider emotions as important for everyday life may improve adolescent’s well-being. This is a new issue that could open to further studies. Exploring meta-emotional beliefs can give us a new insight into how people in general and adolescents in particular live their emotional sphere and to what extent they give attention to emotion in everyday life.

The third group of results in our study refers to the association between EI and sociometric status. Our results failed to evidence an association between self-concept about emotional abilities and acceptance or rejection by peers. In this sense, our results are not consistent with previous studies (Petrides et al., 2006; Mavroveli et al., 2007; Zavala et al., 2008; Andrei et al., 2015) that, however, investigated trait or mixed EI. Our results are not consistent also with the study by Mabekoje and Ogunyemi (2003) that used the self-report scale by Schutte, that is theoretically more similar to the emotional self-concept scale used in the present study, since they are both inspired by Mayer and Salovey (1997) model. However, Mabekoje and Ogunyemi (2003) involved in their study late adolescents (17 years old) and the age of participants could be again a possible source of difference from our study. Indeed, it is simply possible to claim that the most of pre-adolescents involved in present study are not able to evaluate themselves since have not yet developed a reliable self-concept about their emotional abilities.

This interpretation is also demonstrated by the evidence that, on the contrary, ability EI results the most important variable predicting acceptance by peers, indicating that pre-adolescents who show high levels of emotional abilities receive a higher number of nominations for acceptance and a lower number of nominations for rejection. Thus, emotional abilities are very involved in social acceptation/rejection, even when pre-adolescents are not aware of them.

This does not mean that awareness is not important: rather, further results indicate that, independently from their emotional abilities, those pre-adolescents who possess adequate meta-emotional knowledge (being able to evaluate their own abilities) receive more nominations for acceptance and fewer nominations for rejections in sociogram, with respect to their schoolmates who are less able to evaluate their own emotional abilities.

Similarly, adolescents who are more accurate in meta-emotional self-evaluation show higher levels of sociometric status, with higher levels of acceptance and lower level of rejection. Low levels of meta-emotional self-evaluation is, in fact, the best predictor of rejection from peers.

Our results also demonstrate that the most “dangerous bias” in evaluating one’s own emotional abilities is the overestimation. Indeed, adolescents who overestimate their emotional abilities in daily life or in testing situations tend to receive fewer nominations for acceptance and higher nominations for rejections in sociogram than adolescents who underestimate their emotional abilities. Probably, overestimators may be victims, in some way, of the well-known Dunning–Kruger effect (Kruger and Dunning, 1999; Dunning et al., 2003): due to their metacognitive flaws, they are not aware of their own emotional difficulties, for instance, in understanding emotional signals expressed by others or in managing emotional situations; then, in interacting with peers, they use their wrong perceptions or inadequate behaviors, and this produce relational problems and less acceptance by others.

Pre-adolescents with concordant meta-emotional profile, on the contrary, independently from their emotional ability: (1) are more attentive, sensitive, and aware of the importance of emotion in everyday life, as demonstrated by their meta-emotional beliefs scores; (2) are more accepted and less refused by others, as demonstrated by their sociometric results.

A further result of the present study refers to the association between eudemonic well-being and sociometric status. As expected, results revealed that eudemonic well-being is positively related to acceptance and negatively related to rejection. To date, we do not know if well-being influence sociometric status or *vice versa*. Moreover, at least in our group of participants, EI does not have a role in this relationship. However, to the best of our knowledge, this is the first study investigating this issue in pre-adolescents and, thus, the simple result of the association among eudemonic well-being and sociometric status may represent an interesting starting point for future psychological literature.

## Conclusion

We are aware that, in general, this study presents some limitations that need to be addressed for in the future. First, the sample size is narrow, all students belonged to the same school and became from similar social context and participant’s age range is quite limited. Altogether, these aspects limit the generalizability of the results. Second, the tool used for measuring emotional and meta-emotional intelligence is new and, so far, has been validated only in the Italian population. Third, as all correlational ones, our study does not allow clarifying the causal direction of the examined relationships. Thus, as already discussed for the relationship between well-being and sociometric status, we cannot say whether adolescents with higher meta-emotional beliefs experience higher well-being since they use emotions in everyday life or if, vice versa, their well-being stimulates them to be more open to emotions. Similarly, it is not clear if pre-adolescents with higher ability EI are able to create better social relationships and thus they are more accepted and less rejected by peers, or rather if social acceptation create the basis for developing and increasing their EI. A similar consideration may be done for meta-emotional variables: in order to develop a meta-cognitive knowledge about their emotional abilities, people need to examine and reframe repeatedly their experiences of success and failure. Thus, it is equally probable that social acceptation may stimulate in adolescents a more accurate reflection on their emotional abilities, but it is also probable that the lack of meta-emotional abilities may cause a loss in sociometric status.

All that considered, it is fair to note that this study allowed gaining an innovative insight on different aspects of adolescents’ EI that have never been explored so far and, in our opinion, are very important to examine in future research. EI is a multi-facet and complex concept and, therefore, it is the time to examine it under different perspectives and point of views, to understand more deeply its role in the other aspects of individuals’ life. The assessment of emotional and meta-emotional profile of adolescents may have also very practical implications: starting from their profiles, it is possible to involve them in tailored emotional educational programs aimed at improve their possible flawless in specifics emotional areas, to promote their awareness about own emotional abilities and to discuss with them the possible cultural misconceptions about emotions that drive their meta-emotional beliefs. Considering the results of our study, this could, in turn, foster their personal well-being and sociometric status.

## Data Availability Statement

The original contributions presented in the study are included in the article/Supplementary Material, further inquiries can be directed to the corresponding author.

## Ethics Statement

At the time of data collection, ethical review and approval was not required for the study on human participants in accordance with the local legislation and institutional requirements. Written informed consent to participate in this study was provided by the participants’ legal guardian/next of kin.

## Author Contributions

AD’A and AG contributed to the study conception and design. Both authors read and approved the final manuscript.

## Conflict of Interest

AD’A developed the IE-ACCME test that is published and commercialized in Italy by Giunti O.S. The remaining author declares that the research was conducted in the absence of any commercial or financial relationships that could be construed as a potential conflict of interest.

## Publisher’s Note

All claims expressed in this article are solely those of the authors and do not necessarily represent those of their affiliated organizations, or those of the publisher, the editors and the reviewers. Any product that may be evaluated in this article, or claim that may be made by its manufacturer, is not guaranteed or endorsed by the publisher.
